# VDBFusion: Flexible and Efficient TSDF Integration of Range Sensor Data

**DOI:** 10.3390/s22031296

**Published:** 2022-02-08

**Authors:** Ignacio Vizzo, Tiziano Guadagnino, Jens Behley, Cyrill Stachniss

**Affiliations:** Institute of Geodesy and Geoinformation, University of Bonn, 53113 Bonn, Germany; guadagnino@diag.uniroma1.it (T.G.); jens.behley@igg.uni-bonn.de (J.B.); cyrill.stachniss@igg.uni-bonn.de (C.S.)

**Keywords:** 3D mapping, 3D surface reconstruction, volumetric integration, TSDF

## Abstract

Mapping is a crucial task in robotics and a fundamental building block of most mobile systems deployed in the real world. Robots use different environment representations depending on their task and sensor setup. This paper showcases a practical approach to volumetric surface reconstruction based on truncated signed distance functions, also called TSDFs. We revisit the basics of this mapping technique and offer an approach for building effective and efficient real-world mapping systems. In contrast to most state-of-the-art SLAM and mapping approaches, we are making no assumptions on the size of the environment nor the employed range sensor. Unlike most other approaches, we introduce an effective system that works in multiple domains using different sensors. To achieve this, we build upon the Academy-Award-winning OpenVDB library used in filmmaking to realize an effective 3D map representation. Based on this, our proposed system is flexible and highly effective and, in the end, capable of integrating point clouds from a 64-beam LiDAR sensor at 20 frames per second using a single-core CPU. Along with this publication comes an easy-to-use C++ and Python library to quickly and efficiently solve volumetric mapping problems with TSDFs.

## 1. Introduction

Robots that are expected to navigate efficiently through real-world environments need maps to orient themselves and to plan paths [1,2,3,4,5]. These maps need to be built from sensor data, and thus robotic systems typically rely on some form of mapping. Nowadays, robots are equipped with various sensors, depending on the size of the environment, application, payload constraints, and budget available. Typically, 3D sensors are a part of such a sensor suite; popular examples are RGB-D cameras or LiDARs. Creating detailed 3D maps from such data sources can be challenging due to the size of a detailed world representation, especially when building high-resolution maps of large areas.

Numerous data structures to realize effective map representations have been proposed [3,6,7,8,9,10,11,12,13,14]. Most of these systems make assumptions about the specific sensor setup and do not provide systems that tackle the problem from a generic point of view. For example, commonly made assumptions about the used sensor often render those systems unsuitable for other sensors. Furthermore, several TSDF fusion systems rely on accelerators, such as graphics processors (GPUs), that may not be available on mobile robots. In this paper, we revisit the problem of creating a 3D map of the environment, trying to make as few as possible explicit and implicit assumptions about the sensor type or the size of the environment to be mapped. We argue that working directly with point clouds, instead of raw sensor data such as RGB-D images or LiDAR range images, makes it possible to realize a mapping system that can handle different types of range sensors.

To this end, we base our system on top of the OpenVDB library [15]. OpenVDB is an open-source C++ library implementing a hierarchical data structure paired with a rich set of tools for the efficient storage and manipulation of sparse volumetric data. The library was originally developed by Museth and colleagues at DreamWorks Animation for rendering films. It offers unbounded volumetric space access, compact storage, and fast I/O operations. Building a robotic mapping system on top of OpenVDB enables us to provide a simple but effective and fast 3D volumetric fusion pipeline without reinventing the wheel.

**Figure 1:**
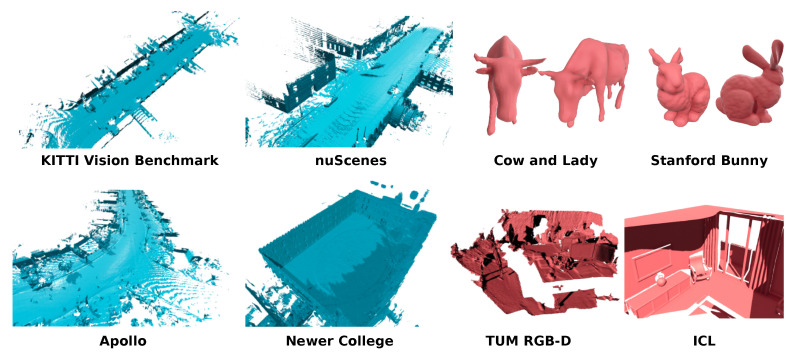
Results of our mapping approach on publicly available datasets showing the versatility of our proposed fusion pipeline. The models colored in light blue corresponds to 3D LiDAR datasets, while the red ones to RGB-D datasets.

The main contribution of this paper is an effective mapping system that comes as an open-source TSDF library and does not require making assumptions about the size of the environment to be mapped. The implementation of our system can be found at: https://github.com/PRBonn/vdbfusion (accessed on 20 December 2021). Our system has been tested on range sensor datasets using different 3D LiDARs and RGB-D cameras but can easily be adapted to other range-sensing modalities. The core algorithm of our system is based on the seminal work by Curless and Levoy [16] and can be realized with our library using a few lines of C++ code. In addition to being simple, our implementation provides excellent results when employed in 3D mapping applications (see Figure 1) while running two to three times faster than state-of-the-art implementations using only a CPU, consuming less memory, and producing compressed map files. Moreover, our system is easy to use, which we showcase by conducting a user study.

Along with this paper, we release a well-designed and carefully crafted C++ implementation with a rich and powerful Python API for rapid prototyping of mapping pipelines. The terminal command pip install vdbfusion is the only command needed to get started. We designed the Python API of VDBFusion to take numpy arrays as input and produce numpy arrays as output, making the library easy to plug into any existing robotics system without dealing with custom data structures. It also supports user-defined data loaders to parse already existing datasets as well as potential future data streams. We also provide a variety of usage examples in both programming languages, C++ and Python. We believe that the practical impact of our VDBFusion future robot mapping systems will be significant.

## 2. Related Work

Mapping is a fundamental part of SLAM pipelines [17,18]. In this work, we focus on the mapping part using 3D sensors providing three-dimensional measurements of the environment. We briefly discuss 3D representations and focus mainly on their use in mapping systems relying on 3D LiDAR sensors or RGB-D cameras.

Over the last three decades, 3D scene reconstruction and understanding have been an active area of research. The first seminal work that relates to ours is a volumetric method for building models from range images by Curless and Levoy [16]. Since then, the use of the truncated signed distance function (TSDF) has gained much popularity in computer graphics, computer vision, and robotics communities. Exploiting the nowadays commonly used low-cost depth sensors, Newcombe et al. [11] popularized with KinectFusion the use of such integration methods [19,20,21,22,23,24,25,26,27,28,29,30,31]. Although numerous systems have been proposed to extend KinectFusion, few of them tackle the problem of mapping environments larger than an office-size room. In this context, Whelan et al. [30,32] introduced one of the first methods that dealt with larger environments by employing a rolling grid that streams out a triangle mesh when exiting the volume being mapped. In this line, sparse data structures have also been explored to build such systems, for example, octrees [1,29,33,34] or voxel hashing approaches [22,24,25,35].

Although such systems show remarkable results, two challenges remained open. First, the above-mentioned systems are hand-crafted for a particular sensor such as Microsoft’s Kinect, often making the implementation unusable for other kinds of sensor modalities. For that, simply consider that it is not trivial to project a $360^{\circ }$ LiDAR scan using a pin-hole camera model. Second, most of these methods work with GPUs, making the mapping method effective but costly in terms of computation and energy requirements. While this is often non-problematic on desktop computers, it often is a limitation for mobile robots with power constraints. Although it might be appropriate for creating maps on a dedicated mobile platform, a mobile robot needs to run mapping pipelines in parallel to localization, obstacle detection, path planning, and other tasks. Thus, the GPU requirement is still a limitation in robotics today.

To cope with this fact, OpenChisel [36] provides an effective solution for the volumetric reconstruction problem on CPU, but it is limited to depth sensors. OpenChisel was later extended by Oleynikova et al.  [24] into Voxblox. To the best of our knowledge, Voxblox is the current state-of-the-art system for building volumetric maps on CPU and is effectively used in numerous systems building upon it [22,27,37]. Additionally, Voxblox is the approach most similar to our work since it is the first of its kind processing point clouds instead of raw depth measurements. Nevertheless, the system can be quite hard to use and virtually impossible to employ outside the ROS ecosystem. This work looks towards a generic volumetric TSDF system that can be deployed in different robotics architectures without necessarily relying on the ROS framework.

With the recent developments in LiDAR technology, numerous mapping systems have been proposed in the SLAM community [38,39,40,41,42,43,44]. Although LiDARs are precise and less noisy than RGB-D sensors, it remains challenging to build efficient 3D maps with such sensors. Wang et al.  [34] proposed a system extending SuperEight [1,29] employing LiDARs. Despite promising results, SuperEight is not publicly available. Additionally, many assumptions need to be made to use that system. Examples include knowing the map size in advance and truncating the LiDAR measurements’ range to a certain range (60 m) to cope with memory and runtime requirements. In addition to these assumptions, SuperEight runs at three frames per second (fps) and is thus slower than the LiDAR sensor frame rate. In contrast, our system can efficiently fuse scans at 30 fps under the same constraints and using the same dataset [45], being, therefore, 10 times more efficient. Lastly, there is currently no information on how this extension [1,34] performs on datasets other than the one used in the original publication [45]. For generating high-fidelity maps, Vizzo et al.  [40] employ Poisson surface reconstruction [46]. Despite the more accurate results obtained compared to TSDF-based methods [22,24], this method is not truly incremental as it requires to buffer around 30 scans before updating its map representations and is therefore not applicable to real-time systems. We showcase the versatility of our system in a variety of publicly available datasets.

Compared with RGB-D systems, extending these mapping systems to the LiDAR domain might seem straightforward. In practice, however, current implementations encode the sensor model inside the algorithm. Therefore, these systems cannot be reused directly for other sensors without a major refactoring process. Moreover, most mainstream open-source libraries for computer vision and 3D data processing such as OpenCV or Open3D provide implementations for volumetric integration. However, none of them work out-of-the-box with LiDAR data. At the same time, a simple and naïve implementation using a dense voxel grid as for RGB-D [11] is simply not possible due to the memory demands. As an example, a small city sequence from the KITTI Odometry dataset [47] that took 2 min to record consumes 30 GB of memory when employing such dense grids. There are mapping systems that build on top of more memory-efficient data structures such as Octomap [6]. Octomap improves the memory consumption notably, but the runtime of its mapping pipeline prohibits the deployment of such method for 3D LiDARs in the real world as it is only capable of integrating such data at 1 fps. In sum, building a 3D mapping system that is memory efficient and fast at the same time remains a challenge. In this work, we aim to fill this gap with the aid of the VDB data structure [15], which was designed for modeling and rendering photorealistic scenes in movie animation.

Inspired by the results of these systems, we aim to develop a robust open-source 3D volumetric integration library that can work with any 3D sensor modality. Additionally, it is developed to be easy to use and extensible to various applications. To implement our system, we build upon the VDB data structure implemented in the OpenVDB [15] library. In line with our work, Macenski et al.  [48] developed a spatio-temporal voxel system for 3D mapping making use of OpenVDB [15]. This system encodes sensor observations into an occupancy grid map that additionally implements voxel decay and decay acceleration [48]. More recently, Besselmann et al.  [49] have also shown promising using the VDB data structure to implement an octree-inspired representation similar to Octomap [6]. While both of these works are closely related to the implementation of our system, the main difference is that we represent the environment as a smooth TSDF surface and the other works represent it as an occupancy grid. Our system can fuse data from LiDARs, RGB-D cameras, or any other 3D sensor, which produces point clouds. As shown in the experiments, our system runs at 20 fps on average for a 64-beam LiDAR and at 10 fps for RGB-D sensors without using GPUs. Our system runs entirely on a single core of a CPU, making our system applicable to being deployed in mobile robots, where power consumption and CPU resources are limited. At the same time, it is highly memory-efficient.

## 3. The VDB Data Structure

When dealing with 3D data such as point clouds in robotics, it is common to employ tree structures, such as octrees [1,2,29,34,50,51]. One of the key reasons behind using such structures is to have virtually unbounded sparse representations of the scene that can be efficiently employed on robotics systems where memory and CPU resources are constrained. Such data structures do not require knowing the size of the environment, where robots might be deployed, in advance.

In line with this, other domains have similar needs. For example, when computing fluid simulations, the volume of the simulation space is not known a priori, as the fluids can virtually expand infinitely. To provide an efficient solution for such applications, the VDB data structure was proposed in the computer graphics community targeting unbounded volumetric data manipulation in the context of creating animated movies.

**Figure 2:**
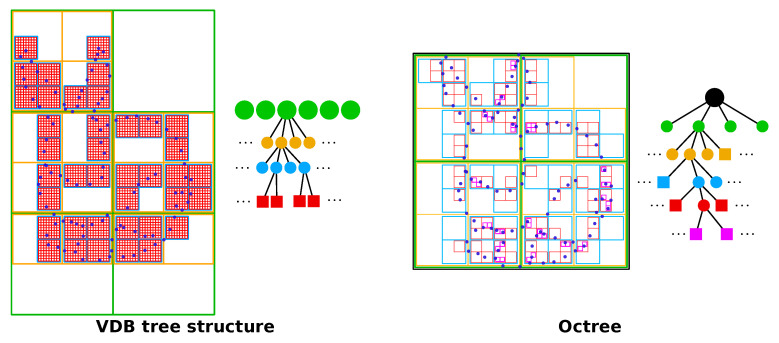
The VDB data structure [15] compared to octrees [52]. A conventional octree subdivides the space increasingly by a factor of 2 on each spatial dimension, starting at a single root node until it reaches leaf nodes (shown as squares) that contain a predefined number of points or size of the octant. In contrast, VDB has a fixed depth with leave nodes that are comprised of 8×8×8 voxels and multiple root nodes (shown as green circles). Due to the fixed depth, access in a VDB data structure is highly efficient compared to traversal in an octree.

The VDB representation is a sparse collection of blocks of voxels (typically 8 × 8 × 8 = 512 voxels) that can be accessed through a hierarchical tree structure with two internal levels, i.e., a fixed height of the involved trees. This height-balanced construction of the VDB results in a shallow and wide representation compared to the less shallow octrees that only have a small branching factor of two on each spatial dimension (see Figure 2). The fixed height of the involved trees allows the implementation of random access algorithms that operate in constant time. Additionally, the representation mimics modern CPU memory architectures with a fixed number of cache levels (L1, L2, etc.) of decreasing size and increasing random-access performance. We invite the curious reader to read more about VDBs and the difference to octrees in the original publication by Museth et al.  [15].

The OpenVDB library [15,53] is an open-source implementation of VDB and has been used in numerous movie production applications over the last decade. OpenVDB is supported by the Academic Software Foundation, which ensures the project’s longevity. Moreover, the fact that the library is still maintained and accepts community contributions allowed us to make slight modifications to OpenVDB to make this publication possible (See: https://github.com/AcademySoftwareFoundation/openvdb/pull/1048 (accessed on 20 December 2021), https://github.com/AcademySoftwareFoundation/openvdb/pull/1055 (accessed on 20 December 2021), and https://github.com/AcademySoftwareFoundation/openvdb/pull/1105 (accessed on 20 December 2021)). Another advantage of using OpenVDB is the large set of tools developed for the library that can be used out-of-the-box. Examples are the OpenVDB visualizer (employed to generate the grid plots shown in this paper), out-of-core storage of grid values, compression of grids, etc.

To the best of our knowledge, OpenVDB is the only well-supported, open source library dedicated to volumetric data applications. Although OpenVDB [15] has been open source for almost a decade, the robotics community paid little attention to it [48,49] in relation to the benefits that the library provides. Potentially, this is rooted in the lack of common vocabulary between communities. As an example, the term TSDF does not appear in the original publication [15], nor does the word “truncated”. To cope with the difference in terminology, we introduce a table of translations, see Table 1, between the common keywords found in VDB applications and robotics. We recommend the reader to inspect the original publication [15] with the table in hand or when inspecting our implementation. In sum, we invite the robotics community to take advantage of the suite of tools associated with VDB that highly match the needs of 3D robotic applications.

To build mapping applications using volumetric data structures, one could potentially adapt existing voxel-hashing systems [1,29,35]. Nevertheless, most existing methods focus on the implementation details of the voxel-hashing approach and require a substantial amount of time start developing a custom application. Additionally, the publications associated with such systems often focus on how the data structures have been implemented, not how to use them. When using our library that builds on top of OpenVDB, the application developer can safely ignore the underlying data structure implementation and use the structure as if it would be a dense voxel grid. The details are handled transparently below the surface.

## 4. The VDBFusion Library for Robotics Applications

In this section, we describe our system for volumetric mapping using truncated signed distance functions [11,16] that exploit VDB via OpenVDB [15]. We describe our design decisions and provide details on the actual implementation and its usage. We kept our system as a mapping library and decoupled it from a SLAM system to be flexible and easy to use in various applications.

### 4.1. System Overview

The goal of our fusion library is to realize TSDF-based mapping [16], allowing us to incrementally fuse data coming from an arbitrary 3D sensor that provides point clouds into a map representation. We should not be required to know the size of the map a priori, and the representation should be memory-efficient, fast to access, and easy to use.

We design our system to process 3D point clouds to achieve this goal. Instead of working with the raw sensor output or a specific sensor model such as the pinhole model for depth cameras, we base our system on 3D point clouds. For every point, we use the location of that point, either in the global or a local coordinate frame, plus the pose of the sensor (position and rotation) when taking the measurement. This pose is available for every 3D point (or point clouds in case no motion distortion occurs). This approach allows us to build a comparably general 3D mapping system.

The input to our system is a set of *N* points $P=\{p_{1},\ldots ,p_{N}\}$, where $p_{i}\in R^{3}$. We expect to know the pose of the sensor when measuring a point. We refer to this pose using the homogeneous transformation $T_{i}\in R^{4\times 4}$. We denote by $R_{i}\in R^{3\times 3}$ and $t_{i}\in R^{3}$ the rotational part and the translational part of the transformation $T_{i}$, respectively. To simplify the description in this paper, we assume here that all points $p_{i}\in P$ are expressed in the global coordinate frame. In our library, however, the user can either provide the points in global coordinates or individual sensor viewpoints together with 3D points in the local sensor frame.

**Figure 3:**
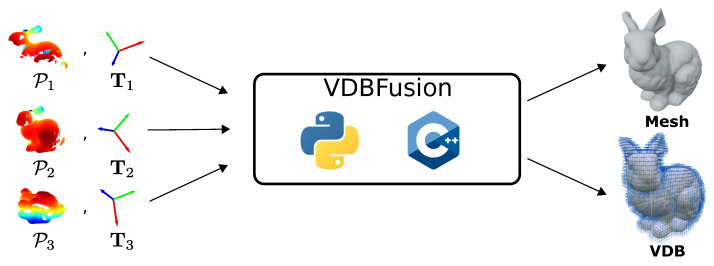
High-level overview of VDBFusion. Our system only takes as input point clouds $P_{i}$ with their corresponding poses $T_{i}$. Based on this information, VDBFusion integrates the sensor data into a sparse TSDF representation, which can be used to compute a triangular mesh representation but can also be used to access the underlying VDB representation.

An overview of the high-level design of our system is shown in Figure 3. The data loader module is in charge of carrying out any projection, pre-processing, or noise filtering as desired. Its implementation is not of relevance to our system. We require the data loader module to output the point clouds in the form of numpy.ndarray or std::vector<Eigen::Vector3d> for the Python and C++ APIs, respectively; analogously, the sensor origin $t_{i}\in R^{3}$ must also be a numpy.ndarray or a Eigen::Vector3d. This assumption is the key ingredient towards a more generic system. Given such point clouds and sensor locations, we can now integrate the data into the TSDF, exploiting the VDB data structure via the VDBFusion approach. After integrating the scans, we can then extract a triangle mesh, individual TSDF values, or the underlying VDB data structure.

To the best of our knowledge, only Voxblox [24] offers a similar fusion pipeline. In contrast to Voxblox, we move all pre-processing to the sensor/dataset-dependent data loader, which performs all pre-processing steps such as minimum range filtering, maximum range filtering, motion undistortion, bilateral filtering, etc. We keep the sensor-data-specific operations in the data loader and outside VDBFusion. This allows us to realize a more elegant fusion pipeline and minimize the number of data-dependent parameters that depend on the employed sensor. As a comparison, our system has only three parameters, while Voxblox requires to set 14 parameters for the mapping algorithm.

### 4.2. Integration Pipeline Implementation

**Figure 4:**
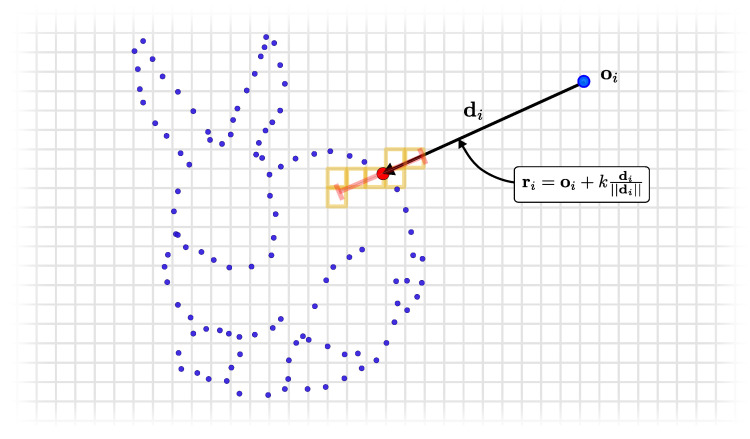
Integration of a measurement (red point) into the global grid representation by ray casting to determine which voxels are passed by the given ray. Only voxels (highlighted in orange) inside the truncation distance (shown in red) are updated.

**Figure 5:**
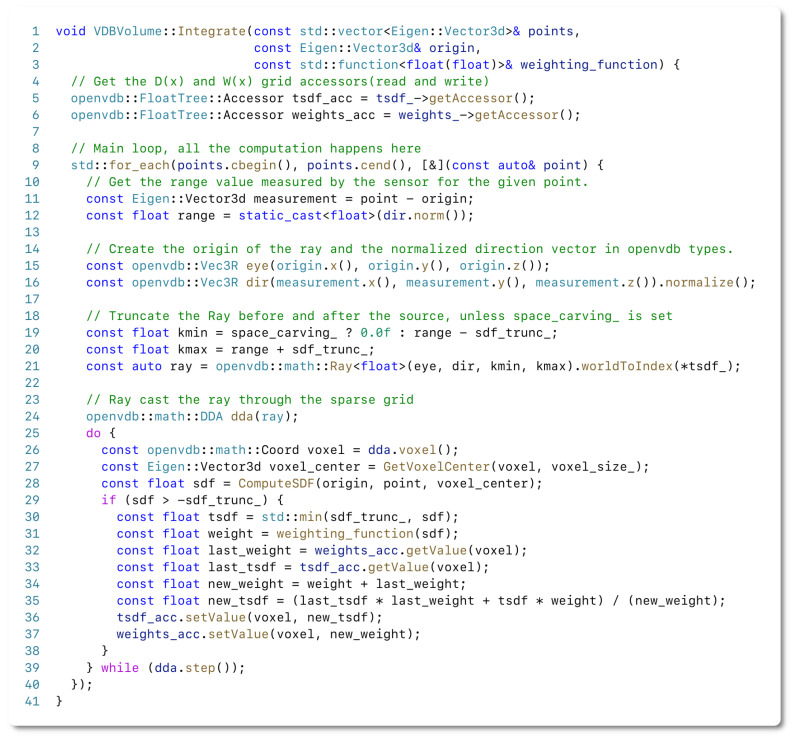
A complete fusion pipeline coded in C++ with a few lines of code.

We follow the approach of Curless and Levoy [16] to integrate point clouds with known sensor location into the current internal map representation represented by a VDB volume. To integrate a new measurement, P, we first need to compute the voxels to be updated in the global grid. To compute the voxel locations $x\in Z^{3}$ in the VDB grid, we raycast a set of rays $R=\{r_{1},\ldots ,r_{N}\}$. Each ray $r_{i}$ is defined by:

(1) $$\begin{array}{cc} r_{i}\left(k\right) & =o_{i}+k\frac{d_{i}}{\left|\right|d_{i}\left|\right|} \end{array}$$

with the origins $o_{i}$ and directions $d_{i}=p_{i}-o_{i}$. The origin of all the rays is at the sensor origin in the global coordinate frame, i.e., $o_{i}=t_{i}$. In line with traditional methods [11,16], we truncate the rays by considering only $k\in [||d_{i}||-\tau_{\mathrm{TD}},||d_{i}||+\tau_{\mathrm{TD}}]$ for fast integration, where $\tau_{\mathrm{TD}}$ is the truncation distance. This process is depicted in Figure 4 and expressed in lines 19–21 of the fusion algorithm shown in Figure 5.

We determine the voxel locations x by the ray–voxel intersections determined via Differential Digital Analyzer (DDA) available in the OpenVDB library. Using the DDA allows us to significantly shrink the length of our integration code and keep it clean using a less error-prone implementation than our own, handcrafted voxel traversal. The use of the DDA is shown in lines 24, 26, and 39 of the code snippet in Figure 5. In Figure 4, the voxel locations x that must be updated are highlighted in orange.

Once the voxels to be updated have been determined, we compute the projective signed distance, $d_{t-1}\left(x\right)$ (line 28 in Figure 5), from the point to the center of each voxel. These signed distance values are then weighted with a weighting function $w_{t-1}\left(x\right)$, we implement this function in the form of a lambda expression passed at runtime (line 31 in Figure 5). The weighted measurements are then integrated into two distinct VDB grids, $D_{t}\left(x\right):R^{3}\to R$, which is a sparse volumetric scalar field representing the signed distances values for each voxel location x, and the weight values $W_{t}\left(x\right):R^{3}\to R$, also in the form of a sparse volumetric scalar field. The integration of these measurements is performed by following the equations introduced by Curless and Levoy [16] representing the TSDF (lines 25–39 in Figure 5):

(2) $$\begin{array}{cc} D_{t}\left(x\right) & =\frac{W_{t-1}\left(x\right)\cdot D_{t-1}\left(x\right)+w_{t-1}\left(x\right)\cdot d_{t-1}\left(x\right)}{W_{t-1}\left(x\right)+w_{t}\left(x\right)} \end{array}$$

(3) $$\begin{array}{cc} W_{t}\left(x\right) & =W_{t-1}\left(x\right)+w_{t}\left(x\right) \end{array}$$

The zero set of the scalar field $D_{t}^{-1}\left(0\right)$ represents the reconstructed surface. It can be computed from the TSDF representation $D_{t}\left(x\right)$ employing techniques based on the popular marching cubes algorithm [54] or by raycasting the TSDF representation $D_{t}\left(x\right)$ [11].

Reading and writing values in sparse data structures are usually the most expensive and hard-to-implement steps for these types of mapping pipelines. By levering the VDB data structure, we efficiently carry out read/write operations in our global map grid. As noted before, we can rely on the implementation of OpenVDB to handle the read/write operations.

We highlight that even when possible, we decided to avoid filthy low-level optimizations of the implementation to make our code more readable for the community. Nevertheless, our not specially optimized but clean implementation is on-par or even faster and more memory efficient than state-of-the-art mapping approaches.

### 4.3. Space Carving

**Figure 6:**
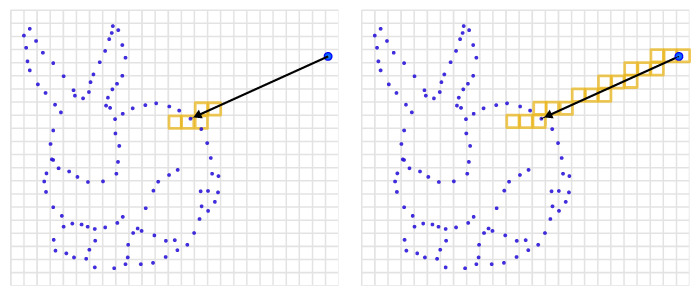
Difference between integration without space carving (**left**) and with space carving (**right**). Without space carving only voxels inside the truncation region are updated, space carving updates all voxels along the ray (colored in orange).

Some mapping applications need to be aware of the free space in the vicinity of the sensor or must distinguish free from unobserved regions. To enable such applications, we also provide space carving on demand. Our approach to space carving modifies the voxels along the ray $r_{i}$ until the measured distance plus the truncation distance is reached, as shown in Figure 6. We truncate it only after the surface by considering voxels in the truncation region, i.e., $k\in [0,||d_{i}||+\tau_{\mathrm{TD}}]$. This will mark all visited voxels as active in the VDB grid with a value that is the same as the truncated value (background value).

Although this might be relevant for mapping applications, it will highly impact the system’s runtime performance. Some valuable conclusions and implementation details about the use of space carving and dynamic object removal are also explored in the experiments.

Note that we do not employ any probabilistic framework, such as occupancy probabilities used in Octomap [6]. We made this design choice with simplicity in mind, envisioning a clean implementation that allows other application developers to extend our system easily.

### 4.4. Weighting

The choice of the weighting functions $w_{t}\left(x\right)$ is not trivial and is extrinsically dependent on the sensor noise and the range of the measurement. Different weighting functions have been extensively studied in the work of Bylow et al.  [19]. Existing implementations have tried to cope with this by providing abstract functions modeling a fixed family of weighting functions [36] or by picking among a variety of hand-picked functions with configuration flags [24]. The use of virtual function typically impacts the runtime of the application, and the weighting functions provided at compilation time [24] might not be required for a given application.

We revise this design choice and employ functional programming, letting the library user pick any arbitrary weighting function at runtime without having to recompile the whole library. The user only needs to provide a lambda expression indicating how to compute the weighting function $w_{t}\left(x\right)$. This enables the faster operation of the integration method and more flexibility when designing a new mapping pipeline for a given sensor modality. For more details on how to use this functionality of our library, see Section 4.7.

### 4.5. Mapping Parameters

As motivated in Section 4.1, we only need a small set of three parameters for mapping since all the sensor-specific preprocessing is performed in the data loader. Therefore, we only have the following three parameters to parameterize the fusion pipeline:

- Voxel size as a floating-point number: The side length of the voxels determines the resolution of the map. The bigger the voxel size, the smaller the map, which comes at the cost of losing high-level details. Analogously, the smaller the voxel size, the higher the level of detail that will be obtained for the final map at the cost of more memory usage and slower runtime operation.
- Truncation distance ($\tau_{\mathrm{TD}}$) as a floating-point number represents the narrow band close to the surface we aim at modeling with the TSDF, i.e., the number of voxels to update in close to the surface. Large truncation distances allow for better smoothing of noise effects of the sensor but make the reconstruction of thin surface challenging due to thickening artifacts and lead to slower runtime operation because it visits more voxels. On the other hand, small truncation distances will lead to a faster runtime operation but are heavily impacted by the sensor’s noise.
- Space carving as a boolean value indicates if space carving should be performed or not. Space carving can effectively remove dynamic objects from the map but comes at the cost of high computational time. In contrast, not performing space carving will lead to higher runtime speeds to the cost of having some dynamic objects artifacts on the map.

While we provide a basic but competitive mapping implementation using TSDFs, our code is relatively easy to extend and improve upon.

### 4.6. Meshing

To extract a triangular mesh from the grid map, we adapted the marching cubes [54] implementation from the Open3D [55] library to work with the VDB data structure. We also extended the implementation to allow filling holes, using the hole filling algorithm described in the work by Curless et al.  [16]. Moreover, we additionally introduce an optional min_weight threshold to extract triangles from the grid only if this matches a given density. For the standard meshing algorithm typically employed with TSDFs, the user can set this threshold to 0. We empirically discovered that similar to PUMA [40], this simple modification enables an out-of-the-box dynamic object removal from the map. The value of the min_weight can also be selected at runtime by the library user.

### 4.7. The VDBFusion Library

We implement our system entirely in C++ and provide a powerful set of transparent Python bindings for efficient and easy usage. In the remainder of this section, we introduce code snippets that serve as starting points for writing a new mapping pipeline using our library. For a complete implementation, we invite the reader to check our MIT-licensed open-source implementation available at https://github.com/PRBonn/vdbfusion (accessed on 20 December 2021).

Most existing approaches require a high effort to get the mapping system up and running. In contrast, we aim at providing an easy-to-use interface. Moreover, we also facilitate the installation of the library; a simple pip install vdbfusion allows the user to get started with the full library. The only Python dependency for the installation is numpy, making our Python package widely portable to different systems and platforms.

We introduce below a draft snippet on using our system for both C++ and Python API. We also provide a rich set of examples together with the source code of our library.

#### 4.7.1. The C++ API

**Figure 7:**
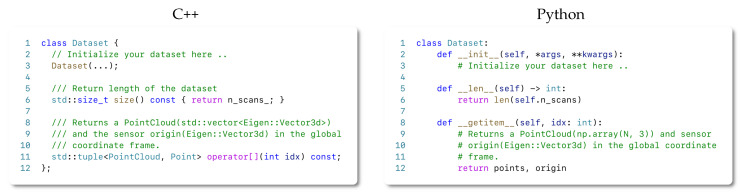
C++ and Python code snippets that implements the suggested 3D data loader code.

**Figure 8:**
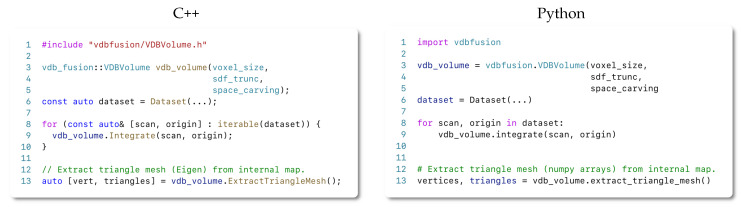
C++ and Python code snippets that implements a fusion pipeline including meshing.

Our thin C++ API only consists of roughly 200 lines of code that enable a powerful yet efficient 3D mapping system. To get started with the library, the user only needs a dataset containing some form of 3D sensor or point cloud data. Although it is not enforced by our library, we recommend creating a data loader module that mimics the one shown in Figure 7. Once the data are ready to be used for our system, a simple C++ or Python application can be written following the structure depicted in Figure 8.

**Figure 9:**
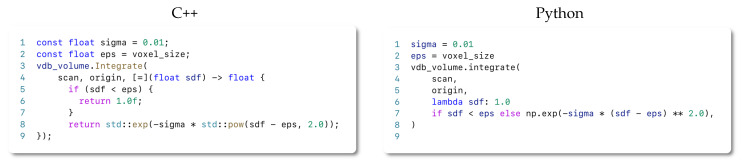
C++ and Python code snippets in how to change the weighting function $w_{t}\left(x\right)$ for TSDF integration. In the example, we implement the exponential weighting function proposed by Bylow et al.  [19].

As described in Section 4.4, if the weighting strategy needs to be changed, one simply needs to pass a function to the integrate method, specifying how to compute the operation. As an example on how to achieve this, we demonstrate how to realize the exponential weighting function introduced by the work of Bylow [19] in Figure 9. We realize the code in the form of a lambda expression.

#### 4.7.2. The Python API

Python has become the most popular programming language for prototyping nowadays. To leverage this popularity to expose our framework to a larger community, we provide a transparent and easy-to-use Python API, which reflects the same functionality as the C++ API. As we show in our experiments choosing Python instead of C++, this choice does not impact the performance. Thus, we are making the selection of Python or C++ a matter of preference without any drawbacks concerning the functionality or runtime performance of the mapping pipeline. As shown in previous snippets, both APIs are highly similar.

Similar to the C++ case, the user only needs a 3D dataset in the form of numpy arrays when using Python. We also recommend (but do not require) defining a data loader similar to the one shown in Figure 7.

## 5. Experiments

The experiments are designed to illustrate the abilities and flexibility of our approach. They showcase that our mapping pipeline is easy-to-use, flexible, fast, and memory—as well as disk-efficient. We furthermore provide comparisons to existing open-source systems. We remark that we only compare our system against existing methods that can process both LiDAR and RGB-D data. As a result of this choice, we skip the comparison to existing RGB-D-only systems such as KinectFusion that only work for camera data. We also remind the reader that our targets are indoor and outdoor environments, potentially large in spatial extent.

We run all the experiments on a CPU without multithreading. The motivation behind this choice is to analyze the system’s capabilities under consideration in addition to the threading model implemented. We tested all methods on a GNU/Linux 64 bits system with GCC 9.3.0, the processor was an Intel Xeon W-2145 with 8 cores @3.70 GHz, and the system had 32 GB RAM. We explicitly paid attention to compiling all baselines enabling optimizations.

To evaluate our system, we pick two datasets, one containing LiDAR data and one with RGB-D data. For the LiDAR dataset, we use the KITTI Odometry benchmark [47], and for the RGB-D, we choose the Cow and Lady dataset [24]. From the KITTI benchmark, we sample sequence 07, a small urban-like sequence with few dynamics objects. The reason behind this choice is that some baselines are not able to map bigger sequences efficiently and thus make some experiments not executable.

For KITTI, we use a voxel size of 10 cm for all integration methods. The maximum usable LiDAR range is 70 m, and the minimum range is 2 m. For the experiments using RGB-D data, we process all points within the range of 0.1 m and 5 m and use a voxel size of 2 mm. The truncation distance is three times the voxel size in all cases.

As baselines, we use the popular Voxblox [24] and Octomap [6] approaches. To the best of our knowledge, these are the only two systems that have an implementation available and that can effectively map both LiDAR and RGB-D data without having to modify the implementation. Unless explicitly stated, all methods are evaluated using their C++ implementations. We compile both baseline methods from the source with all optimizations enabled. We do not make use of potentially provided ROS wrappers.

The main idea behind the experimental section is to put all three systems under test and benchmark runtime performance, memory usage, disk usage, and mapping accuracy. We systematically employ the same experiment independently of the sensor modality of the dataset and show the ease of use for our system.

### 5.1. Runtime

The first experiment is designed to evaluate the runtime performance. It is a common practice to evaluate this experiment iteratively, integrating synthetic scans and analyzing the statistics of the results. For this evaluation, we use a sequence from the KITTI Odometry Benchmark dataset. Sequence 07 is an urban driving sequence. We integrate all the scans on all the internal map representations and then average the runtime of the whole sequence to estimate the results.

We use the Google benchmark suite to compute the results. We explicitly stop the timers when data are being loaded or when some data conversion is being performed. This provides a fair comparison and guarantees that all results reflect how much time it takes for one of the systems to integrate new scans into the internal map representation. We also distinguish between the mapping approaches, running with and without space carving. Naturally, those who make use of space carving will be slower than their counterparts.

The baseline methods evaluated in this experiment are Voxblox and Octomap. Since Octomap does not support an integration method without performing space carving, we skip this baseline when considering no space carving.

It is also important to remark that depending on the mapping application, space carving might be required or not. As we show in this experiment, including space carving will heavily impact the runtime of the mapping system. We state that a simple integration method with no space carving could also yield good results with the additional benefit of having a faster runtime, although not without losing the empty vs. unseen space information in the map. This technique might also impact the dynamics of the final result.

As can be seen in Table 2, our system can integrate LiDAR data at roughly two times the standard sensor frame rate, making it a ready-to-use system in real applications. Additionally, we are 2–3 times faster than the baselines. While Octomap can effectively cope with dynamic objects in the scene, its runtime makes this system difficult to deploy on real-world applications that require high sensor frame rate integration.

While Python is commonly thought of as a slow language, for our implementation, we spent extra effort to make the interoperability between C++ and Python as transparent and efficient as possible. The motivation behind this choice is that nowadays, Python is a common choice for prototyping systems and gives a fast entry point to the library. To asses the runtime performance of our Python API, we use the same datasets as before. As seen in Table 3, our Python API is on par with the C++ implementation in terms of integration speed. The other baselines do not provide official Python implementations, so we skip Octomap and Voxblox for this experiment.

### 5.2. Memory Efficiency

The second experiment analyzes memory usage during mapping. It illustrates that our system does not have an excessive memory consumption, even for mapping large scenes. Note that the VDB data structure also provides other out-of-the-box possibilities to cope with even larger environments than the ones we study in this publication, including out-of-core value storage, where the topology of the grid is stored in RAM, but the values can be offloaded to a hard drive. We do not consider such memory optimizations in this experiment since none of the baselines have similar capabilities. Voxblox does not provide any means to compute the memory usage of the internal map representation, and therefore we skip this baseline for evaluation. To carry on this experiment, we proceed as in Section 5.1 and process the entire sequence 07 of the KITTI Dataset and the Cow and lady dataset. We also provide the in RAM consumption of more naïve mapping approaches, namely, point clouds and dense voxel grids. We do not carry out any particular experiment to obtain these values but rather compute the memory usage since it is deterministic. For point clouds, each point consumes three times the size of a floating-point value, and for dense voxel grids, we compute the bounding box of the resulting map and then fit a regular dense voxel grid in it.

As shown in the Table 4, for the case of the LiDAR sensor modality, the usage of point clouds or dense voxel grids as the map representation is virtually impossible. In contrast, Octomap requires less memory to represent the map, but VDBFusion shows overall the smaller memory footprint. When using RGB-D data, the difference between the memory footprint of the dense voxel grids, Octomap, and VDBFusion is not as pronounced.

### 5.3. Disk Usage

One aspect of a mapping pipeline is its capability to store the map efficiently, and in this experiment, we evaluate the disk footprint of VDBFusion compared to other options. We compare the size of a point cloud map, which is still a popular choice despite its file size requirements. For this, we aggregate all the point clouds into a global coordinate frame and export the result to a binary file format using the Open3D library. Storing the raw point clouds is the upper bound in disk space consumption.

The VDB data structure also supports lossless compression out-of-the-box. This allows for an efficient reduction in the size on disk, especially attractive for very large scenes. Likewise, Octomap also supports an optimized serialization protocol, which we use here for comparison. Contrarily, Voxblox does not provide any serialization mechanism; therefore, we use for this experiment the triangular mesh provided by Voxblox as the map representation on disk. We also report the mesh size that can be extracted from VDBFusion to serve as a more fair comparison with Voxblox.

**Figure 10:**
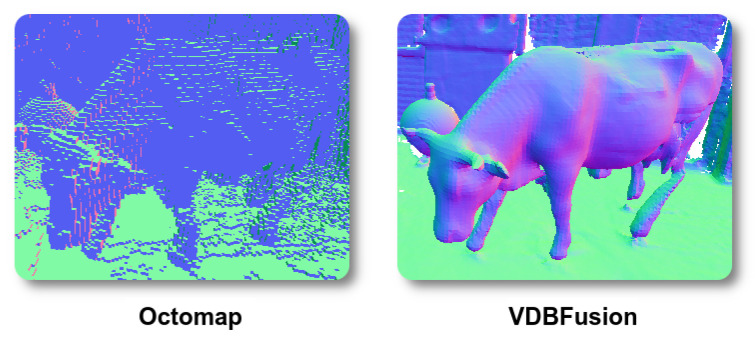
Qualitative comparison between Octomap and VDBFusion. While Octomap clearly models the scene, it does not achieve a high level of detail. Our fusion pipeline shows a higher level of details on the surface when compared with Octomap.

Table 5 shows the size on disk of the different aforementioned options when the resulting maps are serialized and stored. Here, we see that storing the raw point clouds is not a viable option. To store its representation, Octomap discards the per-node probabilities and keeps only the maximum likelihood estimate of the map. In the resulting file, each node occupies only 16 bits of memory. Although this is a highly compressed map, the details of the reconstruction, as shown in Figure 10, are not on par with our results. Furthermore, Octomap cannot integrate new measurements into an existing map once this has been serialized to the disk. The VDB file size is less efficient to store compared to Octomap because it requires a floating-point value for each voxel in D(x) and one floating-point value for each voxel in the weight grid W(x). However, compared to Octomap, the VDB representation on disk enables us to update the map even after it is stored on the disk.

**Figure 11:**
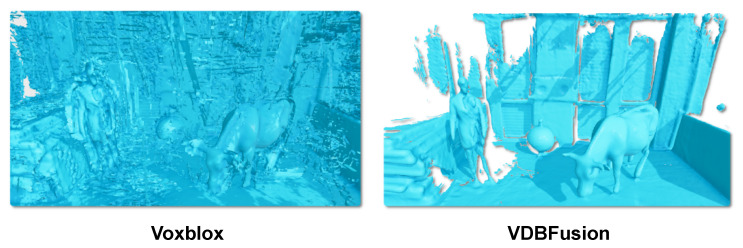
Qualitative comparison between Voxblox and VDBFusion, where we extracted for both representations the triangle mesh from the represented TSDF. While Voxblox shows many artifacts in the background, our fusion pipeline shows a much cleaner surface reconstruction recovering fine details of the scanned scene.

Compared to Voxblox the mesh representation of VDBFusion is much smaller as it contains less artifacts, as shown in Figure 11. Due to the additional artifacts produced by Voxblox, the serialized mesh size tends to be higher than the triangle mesh extracted from VDBFusion. Analogously to the Octomap case, the mesh representation can not be updated after the map has been stored on disk.

### 5.4. Mapping Accuracy

In this experiment, we evaluate the mapping accuracy of Voxblox, Octomap, and our mapping pipeline. To this end, we densely sample the maps generated by Voxblox and VDBFusion into a point cloud. Octomap provides an out-of-the-box method for converting the map representation to point clouds, so we use this one instead for the evaluation. This sampled point cloud is then compared to the reference point cloud. For the case of the KITTI dataset [47], we aggregate all the points in the sequence without downsampling, and we further remove all dynamic objects by using manual annotations from the SemanticKITTI dataset [56]. The Cow and Lady reference point cloud was obtained with a high-resolution scanner, and it is provided along with the original dataset [24]. To sample the point clouds from the mapping baselines. We use an uniform sampling strategy. In the case of the KITTI, we sample 100,000,000 points, and we sample 1,000,000 points for the Cow and Lady dataset. The motivation behind the choice of the number of points to be sampled is to match the density of the reference point clouds used for the evaluation. To assess the performance of the obtained map, we compute the point-to-point distance in meters between the reference point cloud and the sampled models under evaluation. We report the mean average distance between reference-model clouds and standard deviation. A lower metric corresponds to a model that closely models the reference point cloud, while a large point–point distance indicates that the map deviates from the reference point cloud.

Table 6 shows the mapping accuracy results with and without space carving for the different datasets. Generally, the results with space carving are more accurate as it removes dynamics and can further clean up the free space of erroneous measurements or noise.

**Figure 12:**
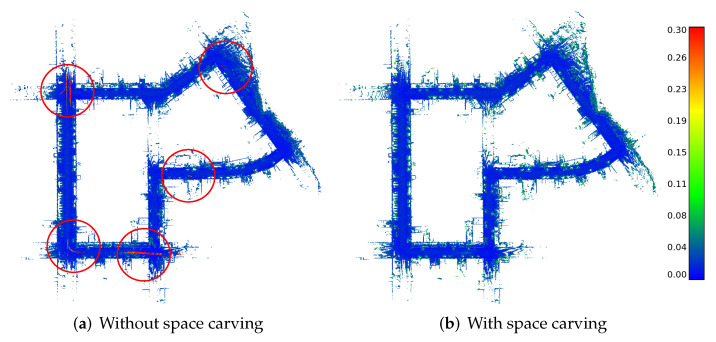
Mapping accuracy results for the KITTI Dataset. (**a**), we show the results without space carving. The reconstructed dynamic objects can be seen by the red color corresponding to a large error (red circles). (**b**), we show the results with space carving removing the dynamics but also parts of the static scene, as visible at the boundaries of the cars.

On the KITTI dataset, we see that our mapping pipeline can produce more accurate maps than Octomap and Voxblox, while the quantitative difference between Voxblox and our pipeline is striking. Figure 12 qualitatively shows the point-wise difference between the ground truth map and the mapping result of our pipeline. Note that mapping without space carving includes dynamics caused by cars and pedestrians (shown by the green/red traces). In contrast, the map with space carving effectively removes the parts corresponding to dynamics. Overall, the point-wise error is very low, i.e., the reconstructed map is very accurate, as indicated by the blue color of the distances.

**Figure 13:**
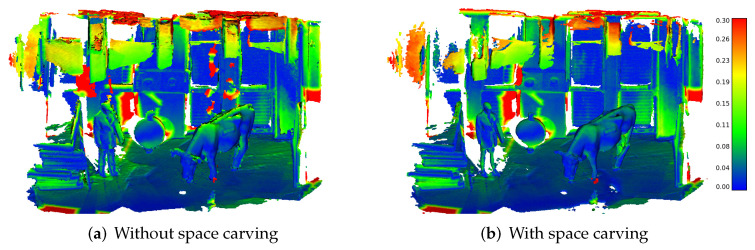
Mapping accuracy results for the Cow and Lady dataset. (**a**), we show the results without space carving with some visible errors on the wall in the back. (**b**) With space carving, these artifacts are effectively removed, and the resulting map is more accurate.

On the Cow and Lady dataset, we can observe that the mapping results of our pipeline are an order of magnitude more accurate than the results produced by Octomap and Voxblox. Figure 11 shows the extracted meshes from Voxblox and our pipeline. We can observe much more artifacts in the extracted mesh of Voxblox. These artifacts explain why the map from Voxblox is a less accurate representation of the environment compared to our reconstructed surface. Figure 13 shows qualitatively the attained point-wise reconstruction accuracy, where we again note that the overall blue color shows the highly accurate reconstruction results of our pipeline.

Overall, the provided quantitative evaluation of the mapping accuracy on indoor and outdoor data shows that our pipeline can reconstruct the different datasets accurately.

Please note that, for the experiments, we used the C++ Voxblox library, although the results do not correlate with the ones shown in the original publication [24]. We suspect that is due to a numeric error in the internal non-standard transformation library. We have contacted the authors to investigate a solution ahead of time, but we could not arrive at one. We also remind the reader that although it would be possible to use the ROS interface, we aim at investigating framework-independent systems; in addition, it requires converting publicly available datasets into ROS (See: https://github.com/ethz-asl/voxblox/issues/373 (accessed on 20 December 2021)).

### 5.5. User Study on the Ease-of-Use

While other approaches claim that they are easy to use or provide a generic and extensive library, we investigate this property by conducting a user case study on the use of our VDBFusion library to provide a quantitative evaluation.

For this, we sampled 10 people from a group of Master’s and Ph.D. students. They all took a robotics lecture at university in the past, but most of the participants did not have any prior experience writing volumetric integration pipelines.

We only provided the participants with the pip package (Python API) and a small set of instructions on how to use it (See: https://www.ipb.uni-bonn.de/html/software/vdbfusion/vdbfusion_user_case_study.pdf (accessed on 20 December 2021)). We did not instruct them on which dataset to use, nor which sensor modality, and we also did not enforce any third party library, since our library only requires numpy to work. We asked the participants to record the times for different steps in the process of generating a map model from the data of their choice. More specifically, we identified the following essential steps needed to get our pipeline running: (1) installing the library, (2) coding the data loader to read the data from disk and provide point clouds as numpy arrays, (3) setting up the fusion pipeline to fuse the data, and, finally, (4) visualizing the generated maps using the provided tools.

**Figure 14:**
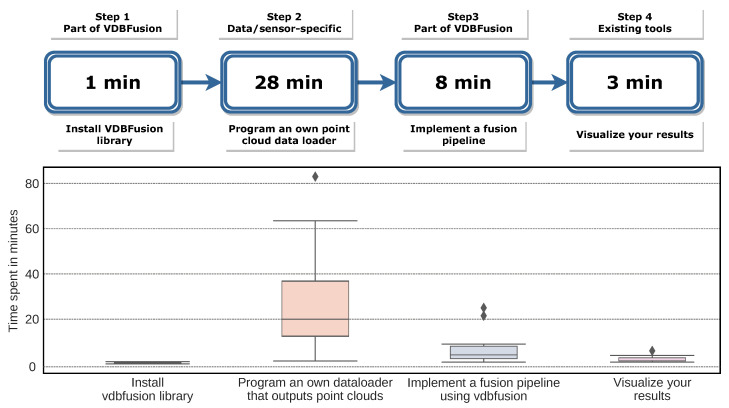
Results of our user study. Here, we show the essential steps and the time needed to perform them as timed by the participants. As can be seen, the most time-consuming part is the writing of a data loader. (**Top**) average time spent per task; (**Bottom**) plot distribution of collected results that highlights the distribution of the data and the outliers. The diamonds indicate the outliers of the distribution.

Figure 14 shows the times reported by the participants with the mean and standard deviation. We notice in this experiment that the most time-consuming task is to write the data loader for the pipeline, i.e., turning the data that the participants have on disk into a point cloud as a numpy array as required by our pipeline. We also highlight that this step is independent of our system; we also note that this step is nowadays a common task for any computer vision and mobile robotics application, meaning that existing data loaders should be available already or can be adapted to our system easily.

As our library is distributed via pip, the installation of the library is only limited by the quality of the internet connection. The installation is achieved via running a single terminal command. Setting up the pipeline is a simple for loop that obtains data from the data loader and calls the integration method. Finally, the visualization of the results is also achieved with a couple of lines of code.

As indicated by the user study, we can attest that the claim of ease-of-use of the provided fusion pipeline is well supported. All participants were able to write a complete mapping pipeline including their data loaders in less than 1 h, on average 40 min, without any external assistance. We could even speed up the process by providing data loaders for most of the commonly used mapping datasets.

### 5.6. Qualitative Results

**Figure 15:**
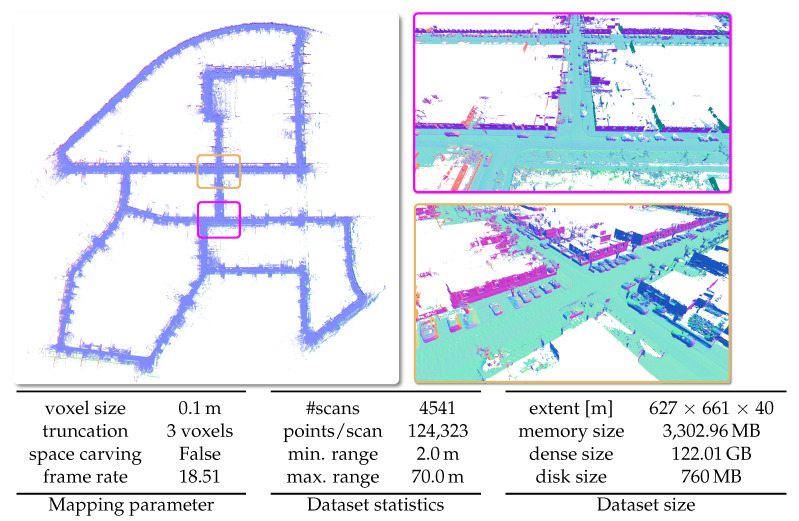
Qualitative results on the KITTI Vision Benchmark [47] with given parameters, dataset statistics, and size of the dataset in respect to spatial extent and memory.

**Figure 16:**
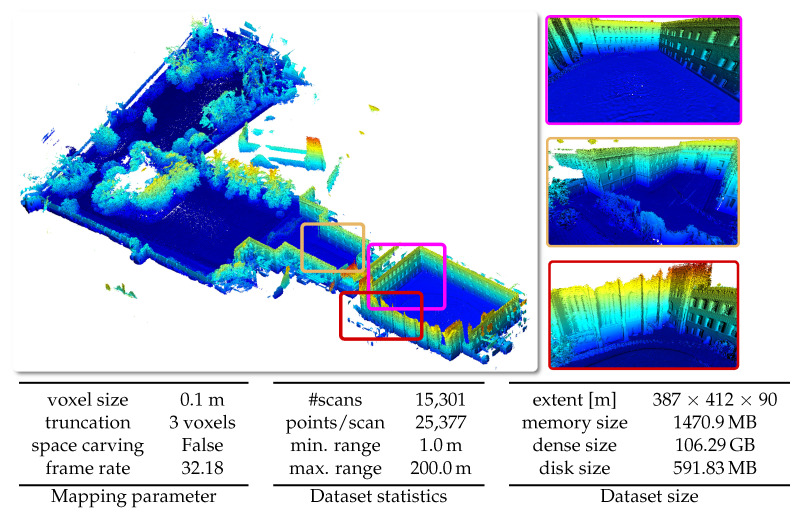
Qualitative results on the Newer College Dataset [45] with given parameters, dataset statistics, and size of the dataset in respect to spatial extent and memory.

**Figure 17:**
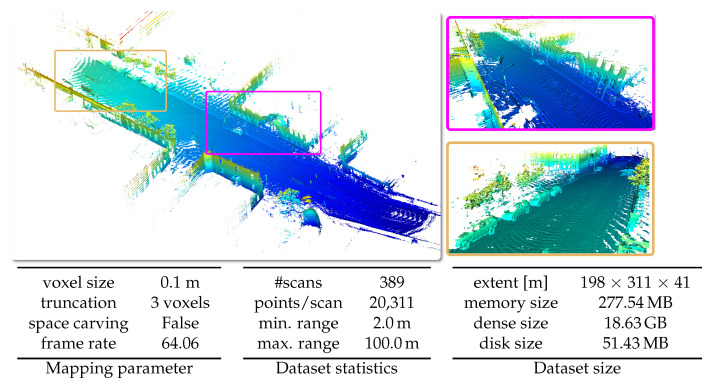
Qualitative results on the nuScenes dataset [57] with given parameters, dataset statistics, and size of the dataset in respect to spatial extent and memory.

**Figure 18:**
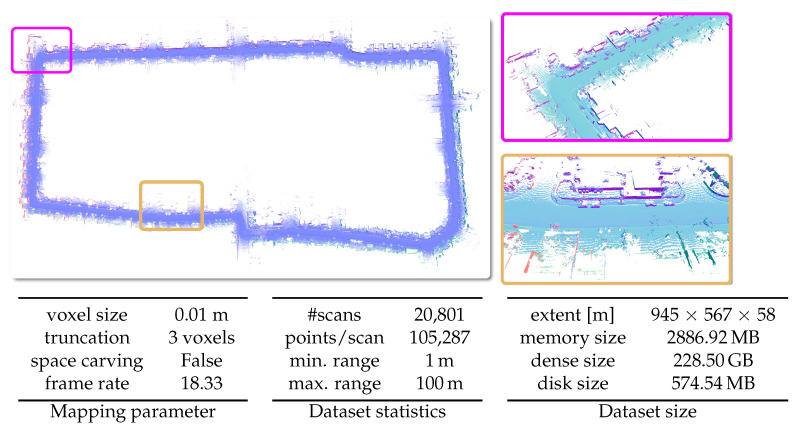
Qualitative results on the Apollo dataset [58] with given parameters, dataset statistics, and size of the dataset in respect to spatial extent and memory.

**Figure 19:**
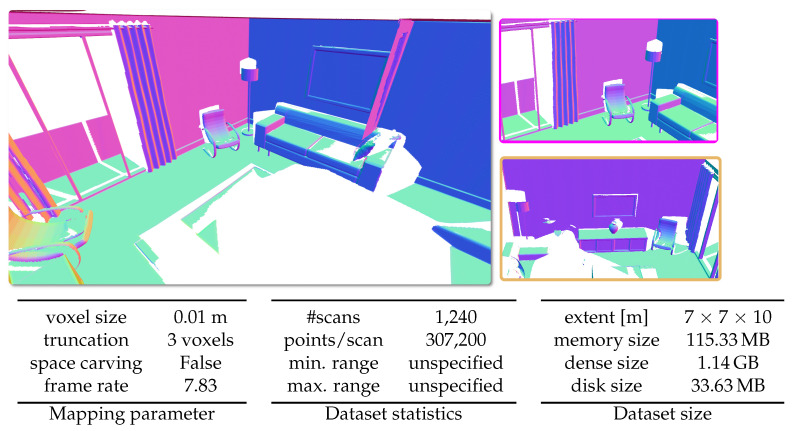
Qualitative results on the ICL-NUIM dataset [59] with given parameters, dataset statistics, and size of the dataset in respect to spatial extent and memory.

**Figure 20:**
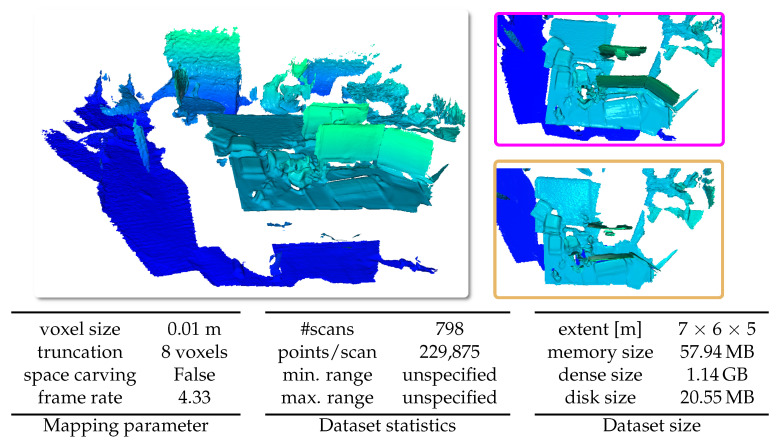
Qualitative results on the TUM RGB-D dataset [60] with given parameters, dataset statistics, and size of the dataset in respect to spatial extent and memory.

In this section, we aim to showcase multiple usages of our system (see Figure 15, Figure 16, Figure 17, Figure 18, Figure 19 and Figure 20). For reasons of brevity, we do not include extensive explanations for the experiments. We only mention the parameters used for the results and some qualitative numbers such as memory consumption, dense grid equivalent, size on disk, etc. In this section, we do not aim to compare against any other baseline but rather show that our system can be applied to multiple domains and different sensors. All necessary codes for these experiments are part of the open-source release. Most of the examples require around  30 lines of Python code, similar to the snippets provided in Section 4.7.

#### 5.6.1. KITTI Odometry Dataset

In Figure 15, we present qualitative results on sequence 00 of the KITTI Odometry Dataset [47]. The dataset uses a 64-beam rotating Velodyne LiDAR sensor mounted on the roof of a car. The dataset provides outdoors scenes of urban, country and highway environments in Germany. The loop-closed poses used to build the map shown in Figure 15 are the output of the 3D-SLAM system SuMa [39].

#### 5.6.2. Newer College Dataset

The newer college dataset was obtained by using a hand-held device through New College, Oxford. For our experiments, we only use the LiDAR data from the 64-beam Ouster sensor used in the system. The poses are the one provided as ground truth poses with the dataset and are not loop-closed. In Figure 16, we present the results of our system using the short experiment sequence.

#### 5.6.3. nuScenes Dataset

The nuScenes dataset is a public large-scale dataset that includes LiDAR data from a 32-beam Velodyne LiDAR sanner mounted on the roof of a car. The dataset was recorded in urban environments at the cities of Boston and Singapore. We exhibit the results of our fusion pipeline on sequence scene-0061. We use the ground-truth poses provided by the dataset.

#### 5.6.4. Apollo Dataset

The Apollo-SouthBay Dataset [58] is a dataset that was collected by driving through different areas in southern San Francisco Bay Area. The point clouds are obtained with a Velodyne HDL-64E LiDAR mounted on the roof of a car, and the ground-truth poses used for the experiment are obtained with the integrated navigation system for data collection. We show the results of our system on the Columbia Park sequence of the dataset.

#### 5.6.5. ICL-NUIM Dataset

The ICL-NUIM dataset is a synthetic RGB-D dataset. It consists of two different scenes with ground truths. For obtaining the result shown in Figure 19, we make use of the Living room scene without the simulated noise. This scene has the depth-maps used in our system, after converting it to point clouds, together with ground truth camera poses.

#### 5.6.6. TUM Dataset

The TUM Dataset [60] is a large dataset containing RGB-D data and ground-truth data. The dataset contains the color and depth images of a Microsoft Kinect sensor along the ground-truth trajectory of the sensor. The data were recorded at a full frame rate of 30 Hz and sensor resolution of 640 × 480. We test our system on the TUM dataset and display the qualitative results of the freiburg1_xyz sequence in Figure 20.

## 6. Conclusions

In this paper, we propose an approach for volumetric mapping based on TSDF representation that exploits a readily available efficient and sparse data structure called VDB. We described our practical implementation that provides an easy-to-use mapping framework that can be used for various sensors providing 3D measurements, such as RGB-D or LiDAR sensors. To handle different kinds of data, we argue that working directly with point clouds makes it possible to use a common mapping pipeline. To this end, we use sensor/dataset-specific data loaders that prepare the data in a way that our mapping pipeline can consume it. Experiments on runtime and memory efficiency show that our implementation is more efficient than other open source mapping frameworks supporting LiDAR and RGB-D data and provides high-quality maps. The evaluation of the mapping accuracy reveals that our approach is more accurate than another TSDF-based pipeline. Lastly, we conducted a user study to verify our claim of easy usage. Our library is provided as open source under the MIT license and available in C++ and Python. We hope that our open-source library opens the door for further research by providing a sane starting point for TSDF-based mapping. Our system can be used in practice to build high-resolution mapping systems for mobile robots. The deployment of such systems in the real world is only possible due the high speed of execution of the integration pipeline implemented in combination with the low memory requirements.

## Figures and Tables

**Table 1:** Terminology used in OpenVDB [15] and corresponding terms commonly used in robotics.

OpenVDB	Robotics
Level set	Signed Distance Field; the zero level crossing represents the iso-surface.
Narrow-band	Truncation region close to the surface.
Half-width	Half width of the narrow-band or truncation distance (typically 3 voxels).
Background-value	Implicit value associated to empty voxels (typically the truncation distance).
Transformation	Representing the voxel size, or resolution, but extended to Affine transformation in general.
Differential Digital Analyzer	Similar to Bresenham’s line algorithm applied to a 3D ray intersecting voxels.

**Table 2:** Runtime results for the Kitti Odometry dataset Sequence 07 [47] and the Cow and Lady Dataset [24]. All values are expressed in frames per seconds (higher is better). The best numbers are highlighted in bold font.

Dataset	w/o Space Carving			w/ Space Carving		
	Voxblox	VDBFusion		Octomap	Voxblox	VDBFusion
KITTI 07	10.11 fps	**19.57** fps		0.42 fps	0.60 fps	**1.37** fps
Cow and Lady	4.76 fps	**14.14** fps		**1.05** fps	0.42 fps	0.84 fps

**Table 3:** Python vs. C++ runtime results for VDBFusion, expressed in average frames per second. The best numbers are highlighted in bold font.

Implementation	KITTI 07	Cow and Lady
VDBFusion Python	18.93 fps	13.28 fps
VDBFusion C++	**19.57** fps	**14.14** fps

**Table 4:** Memory consumption of different investigated variants (lower is better). The best numbers are highlighted in bold font.

Dataset	Point Cloud	Dense Voxel Grid	Octomap	Voxblox	VDBFusion
Kitti 07	2.95 GB	30.6 GB	1.12 GB	n/a	**847.0** MB
Cow	8.57 GB	363.5 MB	124.5 MB	n/a	**122.9** MB

**Table 5:** Disk usage of the serialized representation of different approaches (lower is better).

Dataset	Point Cloud	Octomap 0/1 Output	Voxblox Mesh Export	VDBFusion Mesh Export	VDBFusion TSDF Volume
KITTI 07	3.0 GB	17.0 MB	672.0 MB	254.0 MB	170.0 MB
Cow	8.6 GB	1.6 MB	208.0 MB	15.0 MB	47.0 MB

**Table 6:** Mapping accuracy comparisons. We report the mean and standard deviation of the point-to-point distance (in meter) between estimated and ground truth map (lower is better). The best numbers are highlighted in bold font.

Dataset	w/o Space Carving			w/ Space Carving		
	Voxblox	VDBFusion		Octomap	Voxblox	VDBFusion
KITTI 07	failed	**0.031** ± **0.102** m		0.033 ± 0.035 m	0.497 ± 1.991 m	**0.023** ± **0.022** m
Cow	0.236 ± 0.298 m	**0.049** ± **0.065** m		0.195 ± 0.262 m	0.319 ± 0.398 m	**0.045** ± **0.062** m

## Data Availability

All data used in this publication is publicly available.
